# Evaluation of cannulated compression headless screws as an alternative implant for superior pubic ramus fracture fixation: a biomechanical study

**DOI:** 10.1007/s00264-023-05710-3

**Published:** 2023-02-07

**Authors:** Till Berk, Ivan Zderic, Peter Schwarzenberg, Tatjana Pastor, Felix Lesche, Sascha Halvachizadeh, R. Geoff Richards, Boyko Gueorguiev, Hans-Christoph Pape

**Affiliations:** 1 AO Research Institute Davos, Clavadelerstrasse 8, 7270 Davos, Switzerland; 2 Department of Trauma, University Hospital Zurich, Raemistrasse 100, 8091 Zurich, Switzerland; 3 Department of Plastic and Hand Surgery, Inselspital University Hospital Bern, University of Bern, Bern, Switzerland; 4 Department of Gynecology and Obstetrics, Asklepios Clinic Wandsbek, Alphonsstraße 14, 22043 Hamburg, Germany; 5 University of Zurich, Harald-Tscherne Laboratory for Orthopedic and Trauma Research, Sternwartstrasse 14, 8091 Zurich, Switzerland

**Keywords:** Superior pubic ramus fracture, Biomechanics, Cannulated compression headless screw, Artificial bone model, Motion tracking

## Abstract

**Background/purpose:**

Pubic ramus fractures account for the most common types of pelvic fractures. The standard surgical approach for superior pubic ramus fractures (SPRF) is a minimally invasive percutaneous screw fixation. However, percutaneous closed reduction and internal fixation of anterior pelvic ring injuries have high failure rates of up to 15%. The aim of this biomechanical study was to evaluate the stability of SPRF following stabilization with retrograde placed cannulated compression headless screw (CCHS) versus conventional fully and partially threaded screws in an artificial pelvic bone model.

**Methods:**

SPRF type II as described by Nakatani et al. was created by means of osteotomies in eighteen anatomical composite hemi-pelvises. Specimens were stratified into three groups of six specimens each (*n* = 6) for fixation with either a 7.3 mm partially threaded cannulated screw (group RST), a 7.3 mm fully threaded cannulated screw (group RSV), or a 7.5 mm partially threaded cannulated CCHS (group CCS). Each hemi-pelvic specimen was tested in an inverted upright standing position under progressively increasing cyclic axial loading. The peak load, starting at 200 N, was monotonically increased at a rate of 0.1 N/cycle until 10 mm actuator displacement.

**Results:**

Total and torsional displacement were associated with higher values for RST versus CCS and RSV, with significant differences between RST and CCS for both these parameters (*p* ≤ 0.033). The differences between RST and RSV were significant for total displacement (*p* = 0.020), and a trend toward significance for torsional displacement (*p* = 0.061) was observed. For both failure criteria 2 mm total displacement and 5° torsional displacement, CCS was associated with significantly higher number of cycles compared to RST (*p* ≤ 0.040).

**Conclusion:**

CCHS fixation presented predominantly superior stability to the standard surgical treatment and could therefore be a possible alternative implant for retrograde SPRF screw fixation, whereas partially threaded screws in group RST were associated with inferior biomechanical stability.

## Introduction

Fractures of the pubic ramus are among the most common pelvic fracture types and a demanding need for their improved management has been reported [1]. There is a strong body of evidence regarding timing and choice of surgical approach for the posterior pelvic ring [2–4]; however, when addressing traumatic of the anterior pelvic ring, particularly the pubic branches, conclusions in the literature are much more ambiguous. Patients with pubic ramus fractures are known to experience time-consuming and painful mobilization [5]. A high mortality rate has been described in elderly patients and the preinjury level of mobility cannot be reached by many [5, 6]. High incidences of chronic pain (48.4%) following surgery for pelvic fractures have also been described [7]. Clinical studies report that disability and chronic pain are associated with nonunion or malunion of pelvic fractures [8].

Minimally invasive percutaneous screw fixation is one of the standard procedures for treatment of superior pubic ramus fractures (SPRF) recognized for shorter surgery times and reduced blood loss when compared to other fixation methods, such as plate fixation [9–11]. However, percutaneous closed reduction and internal fixation (CRIF) of anterior pelvic ring injuries has witnessed high failure rates of up to 15% [11–13]. Common failures include screw migration and fragment displacement. Alternative percutaneous surgical implants addressing SPRF could be of great benefit for this well validated surgical method to provide reasonable outcomes with potentially reduced implant failure rates.

Therefore, the aim of this biomechanical study was to evaluate the stability of SPRF following stabilization with retrograde placed cannulated compression headless screw (CCHS) versus conventional fully and partially threaded screws in an artificial pelvic bone model. The hypothesis was that CCHS would provide equivalent or greater stability when compared to conventional ramus screws. To our knowledge, this surgical technique of CCHS fixation of SPRF has never been evaluated.

## Materials and methods

### Specimens and fracture model

Eighteen anatomical composite hemi-pelvises (9 left/9 right, model LSS4060/Hard®, Synbone, Zizers, Switzerland) were used in this study. A type II fracture was created in each specimen at the middle zone of the superior pubic ramus according to the Nakatani classification system [11] by means of an osteotomy using a 1 mm sawblade. A custom template was used to ensure that all vertical osteotomies were identical. The inferior pubic ramus was also osteotomized with a 10 mm gap osteotomy. This discontinuity allowed the superior pubic ramus to be isolated from the influence of the inferior ramus, limiting the parameters affecting the investigated region.

### Study groups

The eighteen hemi-pelvis were stratified into three groups of six specimens each (*n* = 6) with equal distribution of left and right anatomical sites for instrumentation as follows:

- Group RST: SPRF stabilization using 7.3 mm partially threaded cannulated screw, length 90 mm (DePuy Synthes, Zuchwil, Switzerland, Fig. 2A)
- Group RSV: SPRF stabilization using 7.3 mm full threaded cannulated screws, length 90 mm (DePuy Synthes, Zuchwil, Switzerland, Fig. 2B)
- Group CCS: SPRF stabilization using 7.5 mm partially long threaded cannulated CCHS, length 90 mm (DePuy Synthes, Zuchwil, Switzerland, Fig. 2D)

### Surgical procedure

**Fig. 1:**
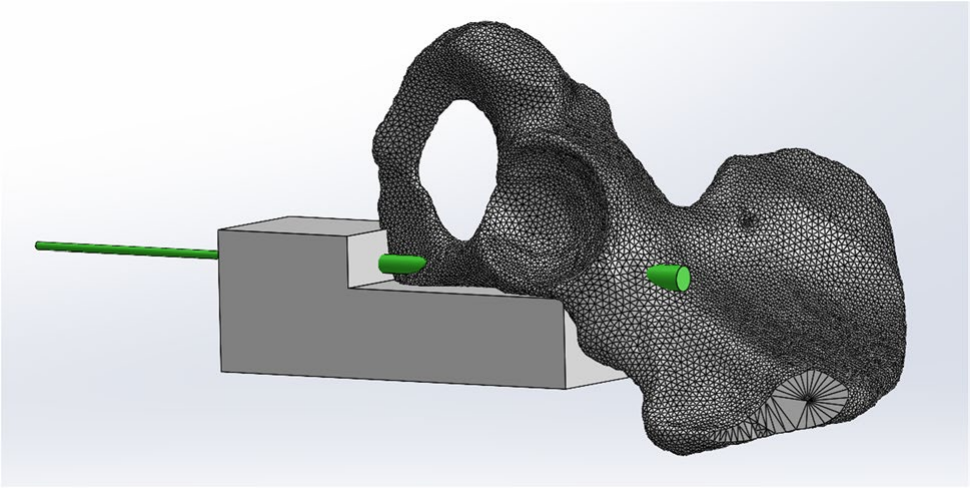
Custom 3D printed aiming device for K-wire placement

**Fig. 2:**
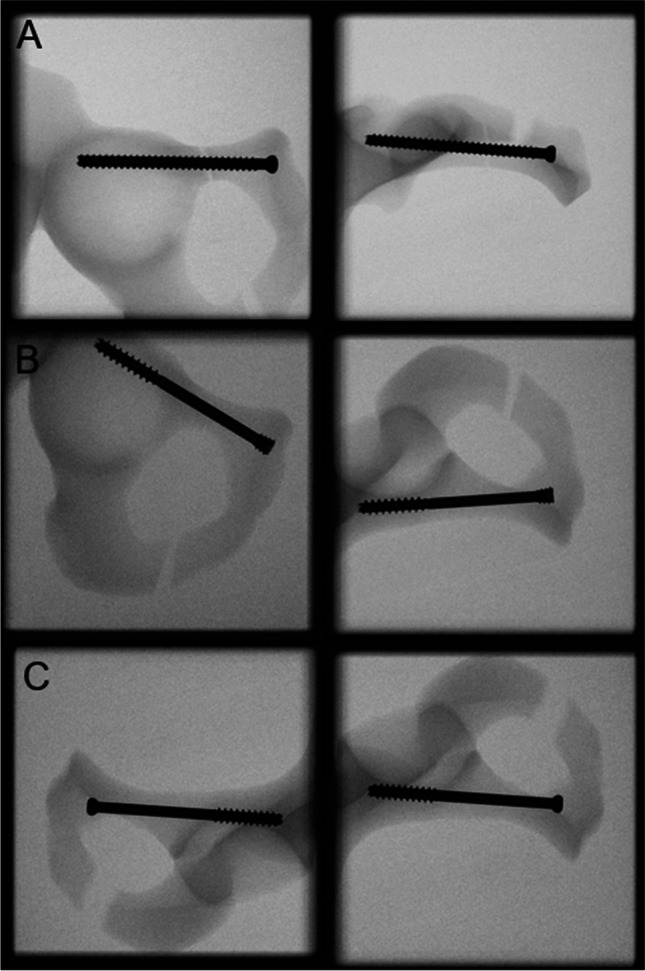
X-rays after instrumentation showing exemplified specimens from group RSV (**a**), CCS (**b**), and RST (**c**)

The surgical treatment of the SRPF was carried out according to the AO principles [14]. Following anatomical reduction of the SPRF, a corresponding 2.8 mm guide wire was placed in the superior pubic ramus across the fracture and cephalad to the acetabulum according to the AO surgery references in a retrograde fashion [14]. The starting point for drilling was the pubic tubercle, following the medial cortical boarder under avoidance of the acetabulum. A custom 3D printed aiming device was employed to ensure that repetitive corrections of the Kirschner-wire placements would not be necessary and possibly affect the outcome. This led to a standardized and repeatable wire and screw placement, ensuring avoidance of any perforations, via falsa, or cortical disruptions. The aiming device allowed the Kirschner-wire to be safely placed in the ideal position in one attempt (Fig. 1). Following pilot drilling, the cannulated screws were placed by inserting them over the K-wire and tightening them according to the operator’s best practice. Inlet and obturator outlet views were obtained with a C-arm, confirming proper screw placements (Fig. 2).

The screws in groups RST and RSV were made from stainless steel (316 L), while those in group CCS were made from titanium alloy (Ti-6Al-4 V). An experienced surgeon with senior consultant status performed all procedures.

### Biomechanical testing

**Fig. 3:**
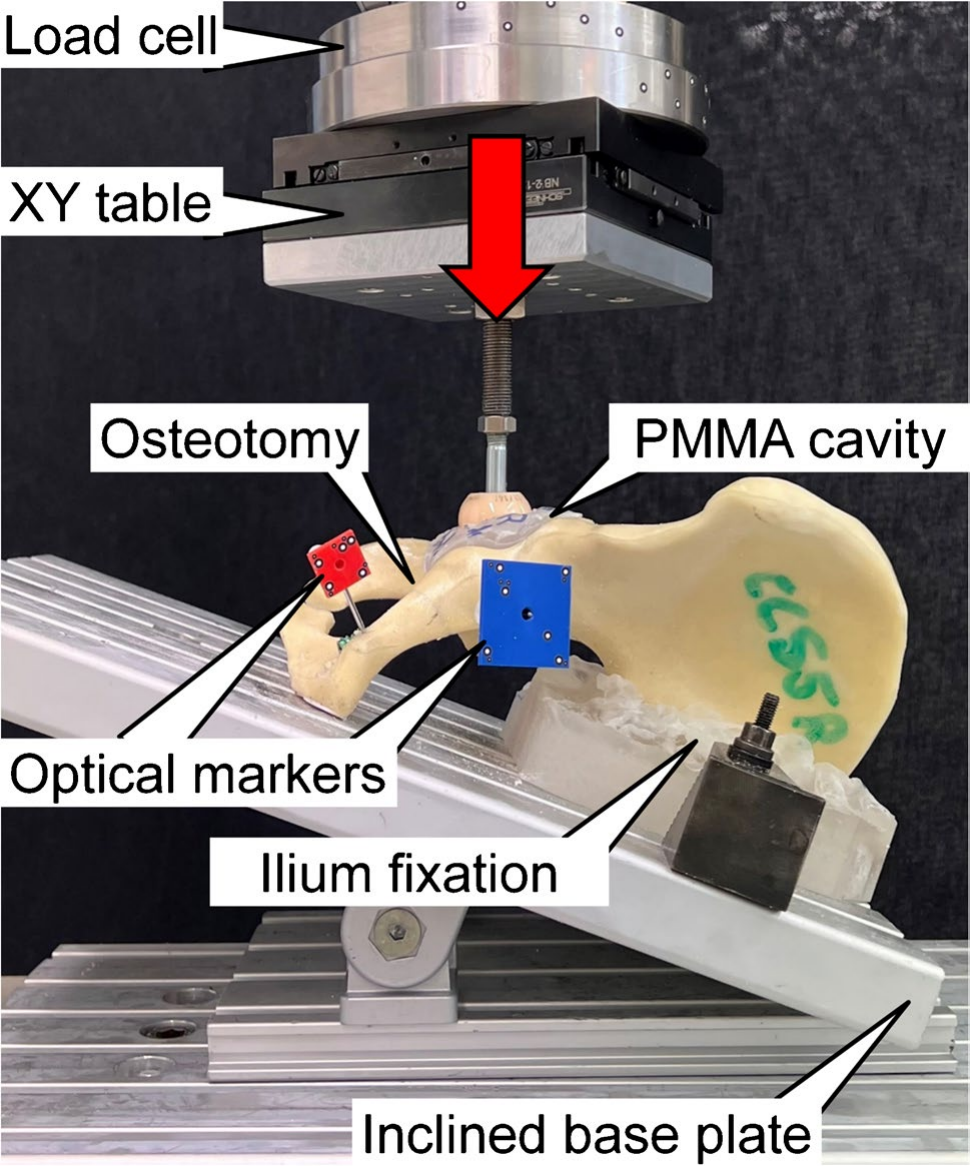
Test setup with a specimen mounted for biomechanical testing

Biomechanical testing was performed on a servohydraulic material test system (Mini Bionix II 858; MTS Systems, Eden Prairie, MN, USA) equipped with a 4 kN load cell (HUPPERT 6, HUPPERT GmbH, Herrenberg, Germany). The setup used to mount the specimen for testing was adopted from previous studies investigating ramus fracture fixation [15] (Fig. 3). Each hemi-pelvic was aligned and tested in an inverted upright standing position. For that purpose, the specimen rested on an aluminum base plate, which was rigidly connected to the machine base, and inclined by 20° in the coronal plane, following the procedure according to Morosato [16] for positioning of the medial aspect of the symphysis and the sacroiliac joint flush with the base plate. The sacroiliac joint was additionally constrained to the base plate via two molded polymethylmethacrylate (PMMA, SCS-Beracryl D-28, Suter Kunststoffe AG/Swiss-Composite, Fraubrunnen, Switzerland) blocks, which allowed consistent mounting of the specimens.

Axial compression along the machine axis was applied to the acetabulum via a ceramic ball of 28 mm radius. Homogenous load transfer to the specimens was achieved by a molded PMMA hemispherical cavity inserted in the acetabulum. The setup configuration targeted a simulation of hip joint reaction force trajectory during walking, as described by Bergmann et al. [17].

The loading protocol commenced with an initial nondestructive quasi-static ramp from 20 N preload to 200 N at a rate of 18 N/s, followed by progressively increasing cyclic loading in axial compression with a physiological profile of each cycle at a rate of 2 Hz [17]. Keeping the valley load at a constant level of 20 N, the peak load, starting at 200 N, was monotonically increased cycle by cycle at a rate of 0.05 N/cycle until the test stop criterion of 10 mm actuator displacement had been fulfilled with respect to its position at the beginning of the loading protocol, which was found adequate to provoke catastrophic failure of the specimens [18, 19].

### Data acquisition and analysis

Machine data of axial displacement and axial load were continuously acquired from the machine transducer and load cell throughout the tests at 200 Hz. Based on these, the construct stiffness was calculated from the ascending load–displacement curve of the initial quasi-static ramp within the linear loading range between 80 and 180 N.

The relative movements between the fractured fragments were continuously assessed at 20 Hz throughout the tests in all six degrees of freedom by means of optical motion tracking. For that purpose, an individual marker set, consisting of multiple single optical markers, was attached to the superior and inferior fragment adjacent to the fracture line using Kirschner (K-) wires. A local anatomical coordinate system was created based on proper alignment of the superior marker set. A second coordinate system was created based on the fracture plane, which was virtually defined using a dedicated touch probe. The coordinates of the markers were tracked with a stereographic optical camera system (Aramis SRX, Carl Zeiss GOM Metrology GmbH, Braunschweig, Germany) within these two coordinate systems. Based on these measurements, the relative movements of the two most anterior aspects—each one associated to either fragment defined by the attached marker sets, but initially overlapping each other at the reduced fracture state—relative to each other, were calculated as the Euclidean normal distance of the translational displacements along the three principal axes and defined as total displacement. In addition, the combined angular displacement was calculated as the gap opening between the two initially reduced osteotomy/fracture surfaces adjoining each other in the fracture gap and defined as gap angle. Furthermore, the angular displacement between the fragments in the fracture plane was assessed and defined as torsional displacement.

The outcome measures were calculated at five intermittent time points of cyclic testing after 2000, 4000, 6000, 8000, and 10,000 test cycles. The latter represented the highest rounded number of cycles at which none of the specimens had failed and dropouts could not artifactually influence the results. The values were considered with respect to the values at the beginning of the cyclic test and were calculated in peak loading condition. Additionally, three different criteria for specimen failure were set at 2 mm total displacement, 5° gap angle, as well as 5° torsional displacement, and the corresponding number of cycles until fulfillment of these criteria were calculated.

Statistical analysis among the outcome measures was performed with SPSS software (v.27, IBM SPSS, Armonk, NY, USA). Mean and standard deviations were calculated for each parameter of interest and group separately. General linear model repeated measures test with Bonferroni post hoc test for multiple comparisons was conducted to determine significant differences between the treatment groups for the outcome measures of the parameters of interest evaluated over the time points during cyclic testing after 2000, 4000, 6000, 8000, and 10,000 cycles. Initial axial construct stiffness and the numbers of cycles for the different failure criteria, including cycles to earliest failure, were compared among the groups with one-way analysis of variance (ANOVA) considering either Bonferroni or Games-Howell post hoc tests. Level of significance was set at 0.05 for all statistical tests.

## Results

Initial construct stiffness was 296.0 ± 50.8 N/mm for group CCS, 365.5 ± 111.1 N/mm for group RST, and 311.3 ± 80.5 N/mm for group RSV, without significant differences among the groups (*p* = 0.411).

The outcome measures for the parameters of interest analyzed over the four time points after 2000, 4000, 6000, 8000, and 10,000 cycles are summarized in Table 1. Total displacement and torsional displacement were associated with higher values for RST versus CCS and RSV, with significant differences between RST and CCS for both these parameters (*p* ≤ 0.033). The differences between RST and RSV were significant for total displacement (*p* = 0.020) and with a trend toward significance for torsional displacement (*p* = 0.061). For gap angle, the outcome measures were homogeneously distributed among the three groups (*p* = 0.972).

**Table 1:** Outcome measures of the parameters of interest evaluated over the five time points after 2000, 4000, 6000, 8000, and 10,000 cycles, presented for each group separately in terms of mean and SD, together with *p* values indicating general significant differences over cycles as well as between the group pairs

Parameter of interest	Treatment	Cycles					*p* value pairwise		
		2000	4000	6000	8000	10,000	CCS	RST	RSV
Total displacement	CCS	0.20 (0.05)	0.38 (0.09)	0.61 (0.18)	0.94 (0.24)	1.33 (0.37)	-	0.021	> 0.999
	RST	0.41 (0.21)	0.78 (0.31)	1.26 (0.41)	1.78 (0.59)	2.88 (1.49)	-	-	0.020
	RSV	0.16 (0.07)	0.37 (0.14)	0.66 (0.26)	0.99 (0.38)	1.40 (0.46)	-	-	-
	*p* value over cycles	CCS: *p* = 0.001; RST: *p* = 0.045; RSV: *p* < 0.001					-		
Gap angle	CCS	0.40 (0.21)	0.81 (0.44)	1.23 (0.65)	1.75 (0.76)	2.32 (1.05)	0.972		
	RST	0.36 (0.21)	0.64 (0.41)	1.00 (0.44)	1.36 (0.53)	2.83 (2.36)			
	RSV	0.37 (0.23)	0.64 (0.40)	1.08 (0.61)	1.66 (0.79)	2.45 (1.41)			
	*p* value over cycles	CCS: *p* = 0.006; RST: *p* = 0.164; RSV: *p* = 0.006					-		
Torsional displacement	CCS	0.68 (0.26)	1.37 (0.32)	2.21 (0.52)	3.54 (0.71)	5.15 (1.27)	-	0.033	> 0.999
	RST	1.66 (0.83)	3.09 (1.18)	4.85 (1.27)	7.25 (2.22)	11.17 (6.74)	-	-	0.061
	RSV	0.53 (0.25)	1.30 (0.59)	2.65 (1.46)	4.22 (2.28)	6.32 (2.96)	-	-	-
	*p* value over cycles	CCS: *p* = 0.001; RST: *p* = 0.081; RSV: *p* = 0.004					-		

**Fig. 4:**
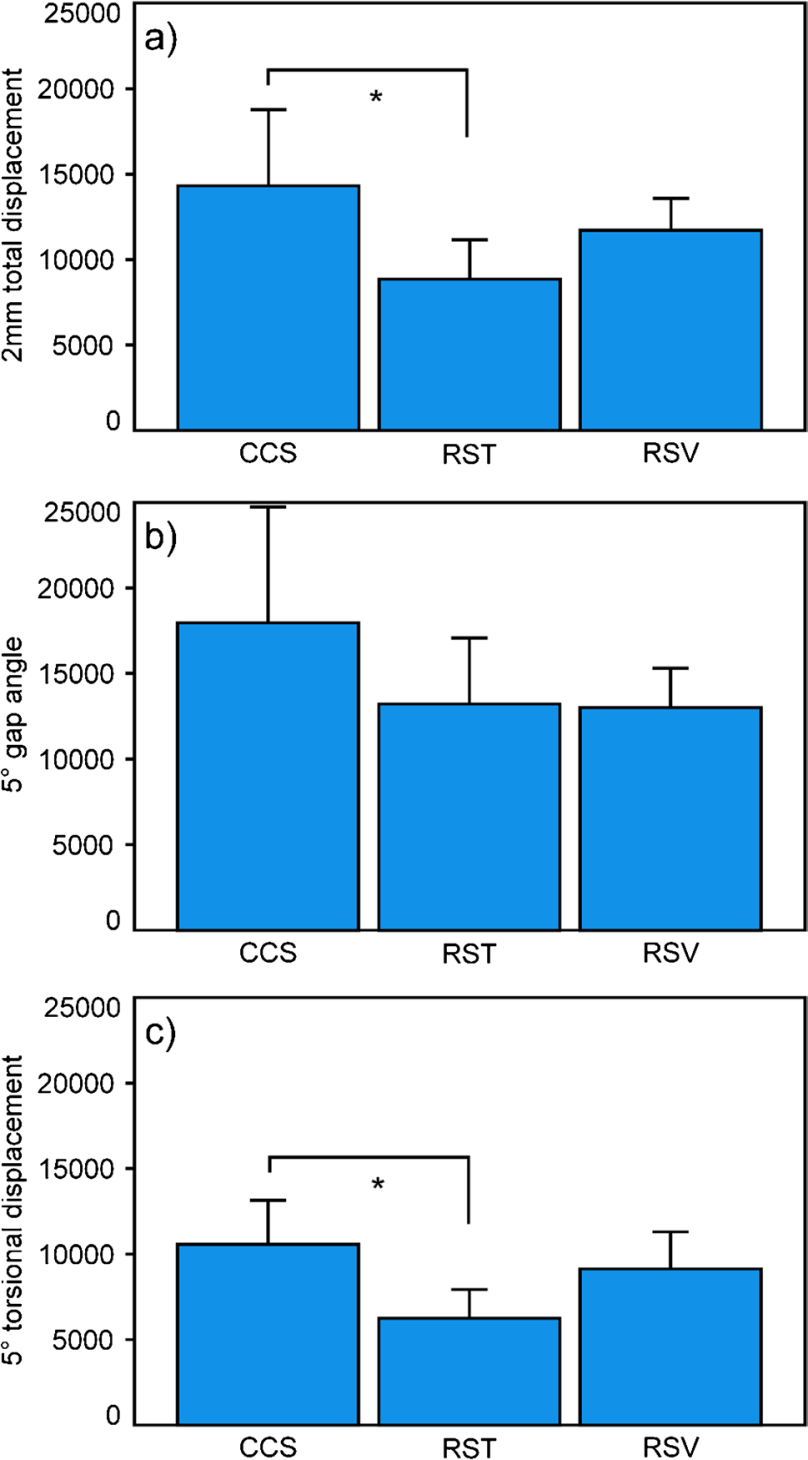
Bar chart presenting cycles to (**a**) 2 mm total displacement, (**b**) 5° gap angle, and (**c**) 5° torsional displacement, shown for each group separately in terms of mean and SD. Star indicates significant difference

The numbers of cycles and the corresponding peak load at the different failure criteria are summarized in Table 2 and Fig. 4. For both 2 mm total displacement and 5° torsional displacement, CCS was associated with significantly higher values compared to RST (*p* ≤ 0.040), with no further significances among the groups (*p* ≥ 0.140). For the criterion 5° gap angle, the values were without significant differences among the groups, *p* = 0.180. Earliest failure unexpectedly occurred by 5° torsional displacement of the fragments relative to each other. The failure modes were predominantly expressed by fracturing of the ilium in between the anterior inferior iliac spine and anterior superior iliac spine.

**Table 2:** Numbers of cycles to and corresponding load at fulfilling the three failure criteria 2 mm total displacement, 5° gap angle, and 5° torsional displacement, shown for each group separately in terms of mean and SD, together with corresponding p values from overall ANOVA output, as well as for pairwise comparisons

Parameter of interest	Treatment	Cycles	Corresponding load (*N*)	*p* value pairwise		
				CCH	RST	RSV
2 mm total displacement	CCH	14,318 (4448)	1631.8 (444.8)	-	0.040	0.526
	RST	8860 (2302)	1086.0 (230.2)	-	-	0.429
	RSV	11,703 (1882)	1370.3 (188.2)	-	-	-
	*p* value ANOVA	*p* = 0.042		-		
5° gap angle	CCH	17,962 (6778)	1996.2 (677.8)	-		
	RST	13,220 (3859)	1522.0 (385.9)			
	RSV	13,000 (2292)	1500.0 (229.2)			
	*p* value ANOVA	*p* = 0.180		-		
5° torsional displacement	CCH	10,572 (2577)	1257.2 (257.7)	-	0.023	0.893
	RST	6254 (1686)	825.4 (168.6)	-	-	0.140
	RSV	9145 (2163)	1114.5 (216.3)	-	-	-
	*p* value ANOVA	*p* = 0.022		-

## Discussion

The aim of this biomechanical study was to assess the stability of cannulated compression headless screws used for fixation of SPRF. Advances on this topic have the potential to reduce the reported high number of implant failures in the literature, while supporting minimally invasive procedures and possibly providing a new implant with greater stability. Outcome measures of the investigated relative interfragmentary movements lead to the following three primary concluding statements.

1. Group CCS demonstrated considerably higher stability in terms of fracture and torsional displacement compared to group RST
2. Although group CCS revealed comparable results to RSV, it was associated with higher stability regarding cycles to predefined failure criteria
3. Group RST was associated with the highest interfragmentary movements and earliest cycles to failure compared to RSV and CCS

According to the AO surgery references, either a fully threaded 3.5 mm/4.5 mm cortical screw, or a 6.5/ 7.3 mm cancellous screw without a washer are recommended for SPRF fixation [14]. A washer does not appear to be an option for the starting point at the pubic tubercle due to the steep and small bearing surface. Due to the proximity to the skin with little overlying muscle tissue, a washer would likely interfere due to its protruding angle. Yet, a washer, presented as an integral component of a screw, has been described as advantageous [20]. It is a standard component of cannulated 6.5 mm or 7.3 mm cancellous iliosacral screw placements of the posterior pelvic ring [21]. Hence, the anatomy-limited absence of the washer may explain the high number of implant failures reported in the literature. The CCHS has threads of different pitch on either end with an unthreaded central part/shaft and is headless. It acts as a countersink, allowing the different threads at each end to draw the fracture fragments together and therefore create compression. This combines the advantages of the screws in group RST and group RSV. The partially threaded screws allow fracture compression, while the fully threaded screws potentially provide more stability due to their proximal thread anchorage. Additionally, it rests flush with the proximal cortex and thus provides less surface for potential irritation. By using the CCHS, both advantages are combined within one screw.

Open reduction and internal fixation of pubic rami fractures using plates are known to result in greater blood loss due to the requirement of wide surgical exposure and can result in severe complications such as injuries to the femoral artery, vena or nerve [22]. Another option for SPRF fixation is an external fixator, which is often used in a polytrauma setting. It is a simple, well-established technique, which is usually applied in a minimal invasive manner and should require the least amount of surgery time. However, the known disadvantages such as percutaneous Schanz-screw related infections and injuries of the lateral cutaneous femoral nerve remain in large numbers [23]. Furthermore, this surgical method always requires a second operation to remove the implants and certainly has the greatest discomfort factor for the patients.

Generally, while using only a single screw, there is always the possibility of fragment rotation around its axis. This seems to be especially present in the smooth, threadless section of a partially threaded screw. In the case of a fully threaded screw, such as the one used in group RSV, intuitively, the rotation should be reduced. This postulation corroborates our finding, since there was a higher rotation in the RST group, yet the differences could not be statistically substantiated as we found no difference between a fully threaded screw and a partially threaded screw.

A biomechanical cadaver study comparing the stability of two 3.5 mm fully threaded screws vs. one 7.3 mm partial threaded screw treating SPRF also presented comparable results [15]. On the contrary, as seen in surgical fixations of ankle fractures, partially threaded screws have been proven to reduce the initial screw stiffness as well as yield load, compared to fully threaded screws [24].

Regarding the entry point, it has been proven that an antegrade screw placement is less likely to result in fixation failure, when compared to retropubic screw fixation [25]. However, antegrade screw placement requires significantly more soft tissue preparation with associated approach morbidities. Yet the chosen investigated fracture in zone II according to Nakatani is most optimally addressed for retrograde screws fixation [11]. Furthermore, biomechanical studies on anterior pelvic ring fractures could prove that retrograde placed intramedullary screw could provide equivalent results to standard plating techniques [26]. Additionally, the retrograde approach for intramedullary screw placements is accompanied with a less excessive surgical exposure.

To address the high rate of reported implant related failure, the authors propose a new implant using secure and minimally invasive well-established techniques to stabilize pubic rami fractures by CCHS. There are known predictors of implant failure such as increased patients age and increased patients BMI, distance and decreased distance from the symphysis have been reported [27]. Increasing patient’s age is typically associated with osteoporosis. The CCHS could bring advantages for this patient group, especially in osteoporotic bone, due to the additional proximal thread locking. This hypothesis could not be addressed in the context of the current study since the data on osteoporotic artificial bone in biomechanics is poor and therefore conventional artificial bone was used.

Further studies on cadaveric bones should be undertaken in the next step to verify if the findings are reproducible in human bones. If the results are comparable, the transition to clinical studies could then be made.

## Strength and limitations

Our results demonstrated a comparable or superior result of the CCHS versus current standard minimally invasive treatment options. However, no screw migration was observed in any of the tests, as seen clinically. The artificial bone seems to have limitations in this regard. Therefore, the used specimen model of artificial bone is the main limitation in this study. However, the authors have performed this first-step investigation because of its novel approach and no available data for comparison. It is further known that artificial bones grant standardized and comparable sample groups, which can overpower the variations in bone quality in human cadaveric specimens and are more cost-effective [28–31]. Synthetic bone specimens have been commonly and successfully used in various previous pelvic biomechanical studies [28, 32–35]. Additionally, the availability of cadavers is limited, leading to a reduced sample size for biomechanical testing as previously reported [36]. Furthermore, the use of artificial bones minimizes the variability of test results between test samples [34]. The chosen sample size in this study was relatively small, nevertheless comparable to related biomechanical studies investigating pelvic fixation techniques [32–35, 37]. Finally, the screws in the CCS group were 0.2 mm wider in diameter than the screws used in the comparison groups (7.5 mm CCHS versus 7.3 mm cannulated screws in Ggroup RST and Group RSV). Since the author’s did not experience any perforation, via falsa, or cortical disruption during screw placements, we believe that this difference can be neglected.

Additional biomechanical studies should be performed using human cadaveric bones and larger sample sizes for a better understanding of CCHS and their potential for the minimal invasive treatment of SPFR.

## Conclusion

The CCHS fixation, introduced in this study, presented predominantly superior stability to the standard surgical treatment and could therefore be a possible alternative implant for retrograde SPRF screw fixation, whereas partially threaded screws in group RST were associated with inferior biomechanical stability.

## Data Availability

The collected data will be stored securely at our institute for 10 years. During this period, they are still available upon request. After 10 years, the data will be deleted; however, all the datasets analyzed or generated during this study will be available from corresponding author upon reasonable request.
